# Endocannabinoid signaling in glioma

**DOI:** 10.1002/glia.24173

**Published:** 2022-03-24

**Authors:** Carlos Costas‐Insua, Manuel Guzmán

**Affiliations:** ^1^ Centro de Investigación Biomédica en Red sobre Enfermedades Neurodegenerativas (CIBERNED) Madrid Spain; ^2^ Department of Biochemistry and Molecular Biology Instituto Universitario de Investigación Neuroquímica (IUIN), Complutense University Madrid Spain; ^3^ Instituto Ramón y Cajal de Investigación Sanitaria (IRYCIS) Madrid Spain

**Keywords:** anti‐tumoral therapy, apoptosis, cannabinoid, G protein‐coupled receptor, glioma, metabolism, tumor microenvironment

## Abstract

High‐grade gliomas constitute the most frequent and aggressive form of primary brain cancer in adults. These tumors express cannabinoid CB_1_ and CB_2_ receptors, as well as other elements of the endocannabinoid system. Accruing preclinical evidence supports that pharmacological activation of cannabinoid receptors located on glioma cells exerts overt anti‐tumoral effects by modulating key intracellular signaling pathways. The mechanism of this cannabinoid receptor‐evoked anti‐tumoral activity in experimental models of glioma is intricate and may involve an inhibition not only of cancer cell survival/proliferation, but also of invasiveness, angiogenesis, and the stem cell‐like properties of cancer cells, thereby affecting the complex tumor microenvironment. However, the precise biological role of the endocannabinoid system in the generation and progression of glioma seems very context‐dependent and remains largely unknown. Increasing our basic knowledge on how (endo)cannabinoids act on glioma cells could help to optimize experimental cannabinoid‐based anti‐tumoral therapies, as well as the preliminary clinical testing that is currently underway.

## Main Points

- Glioma cells express cannabinoid receptors, whose pharmacological activation inhibits tumor growth in preclinical models.
- The biological modulation of the endocannabinoid system in glioma exerts complex, context‐dependent effects on tumor growth.

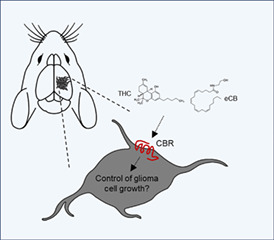

## 1 INTRODUCTION

Gliomas have been traditionally defined as tumors of the central nervous system that display immunohistochemical and ultrastructural evidence of glial differentiation. The WHO now classifies gliomas according to several criteria, including cellular features (i.e., resembling astroglia, oligodendroglia or ependyma), grade of malignancy (i.e., from 1 to 4), and molecular features [e.g., isocitrate dehydrogenase (IDH) or histone H3 status] (Louis et al., 2021). Within the many different types of gliomas, glioblastoma (formerly “glioblastoma multiforme” or “grade 4 astrocytoma”; currently “glioblastoma, IDH‐wildtype” in the strict sense, to distinguish it from “grade 4 astrocytoma, IDH‐mutant”) is the most frequent type of malignant primary brain tumor in adults, and one of the most aggressive forms of cancer. Consequently, median overall survival after diagnosis and benchmark treatment is around 15 months. This dramatic behavior is mainly due to the high invasiveness and proliferation rate of glioblastoma cells. In addition, glioblastoma exhibits a high resistance to common chemotherapy and radiotherapy, which is usually ascribed to the varying mutations frequently found in this tumor that affect key pathways involved in the control of processes as cell proliferation, survival, and DNA repair (Alexander & Cloughesy, 2017; Wen et al., 2020). Among the numerous signal‐transduction platforms that are affected in glioblastoma cells, G protein‐coupled receptors (GPCRs), the largest superfamily of cellular receptors, has gained great attention during the last years (Byrne et al., 2021; Cherry & Stella, 2014). Specifically, glioma cells express endocannabinoid‐sensing GPCRs (i.e., type‐1 cannabinoid receptor, CB_1_R; and type‐2 cannabinoid receptor, CB_2_R), whose pharmacological activation targets multiple cancer hallmarks such as resistance to programmed cell death, angiogenesis, cell proliferation, and cell invasiveness and metastasis (Dumitru et al., 2018; Ellert‐Miklaszewska et al., 2020; Velasco et al., 2012). Here, we will review our current knowledge on cannabinoid receptor‐evoked molecular mechanisms and pharmacological effects in glioma cell‐based laboratory models. As glioma cells used in preclinical research are often of high grade (i.e., grades 3–4), and their IDH status is normally not provided, here, unless otherwise specified, we will include under the term “glioma cells” both “glioblastoma, IDH‐wildtype” and grade 3–4 “astrocytoma, IDH‐mutant” cells. We will also discuss the notion that targeting cannabinoid receptors might be a new strategy to improve therapeutic interventions against glioblastoma in the clinical setting.

## 2 EXPRESSION OF THE ENDOCANNABINOID SYSTEM IN GLIOMA

Preparations from the hemp plant *Cannabis sativa* L. have been used medicinally for millennia. Among their unique active components (the cannabinoids), Δ^9^‐tetrahydrocannabinol (THC) is the most relevant owing to its high potency and abundance. THC exerts a wide variety of biological effects by mimicking endogenous substances [the endocannabinoids anandamide (*N*‐arachidonoylethanolamine, AEA) and 2‐arachidonoylglycerol (2‐AG)] that engage specific cellular cannabinoid receptors (Mechoulam et al., 2014; Pertwee et al., 2010). Endocannabinoids, together with their receptors and the proteins responsible for their synthesis, transport, degradation, and bioconversion, constitute the so‐called “endocannabinoid system” (ECS), a pivotal neuromodulatory network that controls a plethora of biological functions. So far, two major cannabinoid‐specific receptors ‐ CB_1_R and CB_2_R ‐ have been cloned and characterized from mammalian tissues. Most of the effects produced by cannabinoids in the central and peripheral nervous system rely on the activation of CB_1_R molecules located largely on neurons. In contrast, CB_2_R is more highly abundant in immune cells, and may also be present in cells ‐including cancer cells‐ from other origins. The cannabinoid receptor ligands AEA and 2‐AG are synthesized from membrane lipids primarily by the enzymes *N*‐acyl‐phosphatidylethanolamine‐phospholipase D (NAPE‐PLD) and diacylglycerol lipase α/β (DAGLα/β; DAGLα accounting for most 2‐AG production in the adult brain), respectively. Subsequently, AEA and 2‐AG are deactivated mainly by the enzymes fatty acid amide hydrolase (FAAH) and monoacylglycerol lipase (MAGL), respectively (Piomelli, 2003; Katona & Freund, 2008; Castillo et al., 2012).

It has been long known that glial cells express functional CB_1_R and CB_2_R, the former especially in neuroglia and the latter especially in microglia (Stella, 2010). Likewise, many glioma cell lines express detectable amounts of CB_1_R and CB_2_R mRNA and protein (Galve‐Roperh et al., 2000; Lorente et al., 2011; Sánchez et al., 1998; Vaccani et al., 2005). Moreover, a series of studies has analyzed the expression of CB_1_R and CB_2_R, as well as other ECS elements, in specimens from human gliomas (Table 1). However, several inconsistencies between these studies have come out, likely because the limited availability of these samples precludes the achievement of appropriate patient‐population sizes and matched (particularly in age and sex) control specimens. Which sample represents the best internal control is a recurrent question in glioma research. So far, the most accepted procedure is to use adjacent, non‐tumoral tissue if resection is safe for the patient during surgery (Lemée et al., 2013), but this has not been applied to all the studies conducted to date (Table 1). Other inherent limitations, such as demographic bias and sample handling, might contribute as well to the observed differences. Overall, the most consistent finding of these studies seems to be the up‐regulation of CB_2_R expression in high‐grade glioma samples (Calatozzolo et al., 2007; De Jesús et al., 2010; Ellert‐Miklaszewska et al., 2007; Hashemi et al., 2020; Held‐Feindt et al., 2006; Sánchez et al., 2001; Schley et al., 2009; Wu et al., 2012). This is believed to occur mainly in endothelial cells of blood vessels and in infiltrated immune cells, although tumor cells also express this receptor (Hashemi et al., 2020; Held‐Feindt et al., 2006). On the contrary, increases, decreases or no changes of CB_1_R expression in high‐grade glioma biopsies have been reported. When comparing to adjacent, non‐tumoral tissue, CB_1_R was found up‐regulated in two studies (Hashemi et al., 2020; Wu et al., 2012), and the report that showed a decreased CB_1_R density (De Jesús et al., 2010) used membrane preparations instead of whole‐tissue homogenates, thus ruling out intracellular receptors a priori. Unlike CB_2_R, CB_1_R seems to reside mainly on glioma cells, as assessed by co‐localization with the astrocytic marker GFAP (Hashemi et al., 2020; Wu et al., 2012).

**TABLE 1:** Expression of endocannabinoid system elements in glioma (published studies)

Element	Change	Molecule (method)	Control tissue	Reference
CB_1_R	= = ↑ = ↓ ↑ ↑	Protein (IHC) mRNA (Q‐PCR) Protein (IHC) Protein (IHC) Protein (WB) & Binding (GTPγS)^a^ mRNA (Q‐PCR) & Protein (WB, IHC) mRNA (Q‐PCR) & Protein (WB)	Gliomas (grades I–III) Normal brain tissue Diseased brain (*n* = 2) and other gliomas Epileptic brain tissue (*n* = 1) Normal brain tissue Non‐tumoral & normal brain tissues Non‐tumoral tissue	PMID: 11479216 PMID: 16893424 PMID: 18175076 PMID: 19480992 PMID: 20307616 PMID: 22176552 PMID: 32623617
CB_2_R	↑ = ↑ ↑ ↑ ↑↓ ↑ ↑	Protein (IHC) mRNA (Q‐PCR) Protein (IHC) Protein (IHC) Protein (IHC) Protein (WB) & Binding (GTPγS)^a^ mRNA (Q‐PCR) & Protein (WB, IHC) mRNA (Q‐PCR) & Protein (WB)	Gliomas (grades I‐III) Normal brain tissue Diseased brain (*n* = 2) and other gliomas Other brain tumors Epileptic brain tissue (*n* = 1) Normal brain tissue Non‐tumoral tissue & normal brain Non‐tumoral tissue	PMID: 11479216 PMID: 16893424 PMID: 18175076 PMID: 17239827 PMID: 19480992 PMID: 20307616 PMID: 22176552 PMID: 32623617
NAPE‐PLD	↓ ↓	Protein (activity) mRNA (Q‐PCR) & Protein (activity)	Non‐tumoral tissue Non‐tumoral tissue	PMID: 15816853 PMID: 22176552
FAAH	↓ ↓	Protein (activity) mRNA (Q‐PCR) & Protein (activity)	Non‐tumoral tissue Non‐tumoral tissue	PMID: 15816853 PMID: 22176552
DAGLα	=	mRNA (Q‐PCR) & Protein (activity)	Non‐tumoral tissue	PMID: 22176552
MAGL	↓	mRNA (Q‐PCR) & Protein (activity)	Non‐tumoral tissue	PMID: 22176552
AEA	↑ ↓	Lipid (GC/MS) Lipid (LC/MS)	Non‐tumoral tissue Non‐tumoral tissue	PMID: 15816853 PMID: 22176552
2‐AG	= ↑	Lipid (GC/MS) Lipid (LC/MS)	Non‐tumoral tissue Non‐tumoral tissue	PMID: 15816853 PMID: 22176552

^a^ A CB_1_R/CB_2_R‐mixed agonist (WIN‐55,212‐2) was used, so changes could be ascribed to CB_1_R and/or CB_2_R.

Fewer studies have analyzed the expression of enzymes involved in endocannabinoid metabolism, and the levels of endocannabinoids themselves, in human gliomas (Petersen et al., 2005; Wu et al., 2012) (Table 1). While enzymes responsible for the synthesis and degradation of AEA were down‐regulated in samples from glioma patients in both studies, the levels of AEA were inconsistent, with the initial report showing increased AEA and the subsequent one finding decreased AEA in the tumor specimens. Another study found reduced levels of AEA in meningioma samples, as well as in a sole glioblastoma biopsy (Maccarrone et al., 2001). As AEA levels are well known to increase over time in *postmortem*, anoxic brains (Schmid et al., 1995), these discrepancies might reflect differential handling of the samples between studies. So far, to the best of our knowledge, only one study has analyzed the expression of enzymes involved in 2‐AG metabolism. The authors found that while DAGLα levels were similar to those of matched controls, MAGL expression was significantly reduced, in concert with the enhanced levels of 2‐AG in the glioblastoma samples (Wu et al., 2012). This is in line with a previous report showing increased levels of 2‐monoacylglycerols in glioma specimens, although in this study a significant 2‐AG boost was not evident (Petersen et al., 2005). Taken together, these data suggest that human gliomas may have an overactive 2‐AG‐CB_2_R signaling axis, although information regarding most components of the system is scarce and, in some cases, inconsistent. Hence, further research is necessary to clarify which precise cell types within the tumor express the different elements of the ECS, as well as how the dynamic regulation of these proteins and lipids along disease malignancy occurs.

**FIGURE 1:**
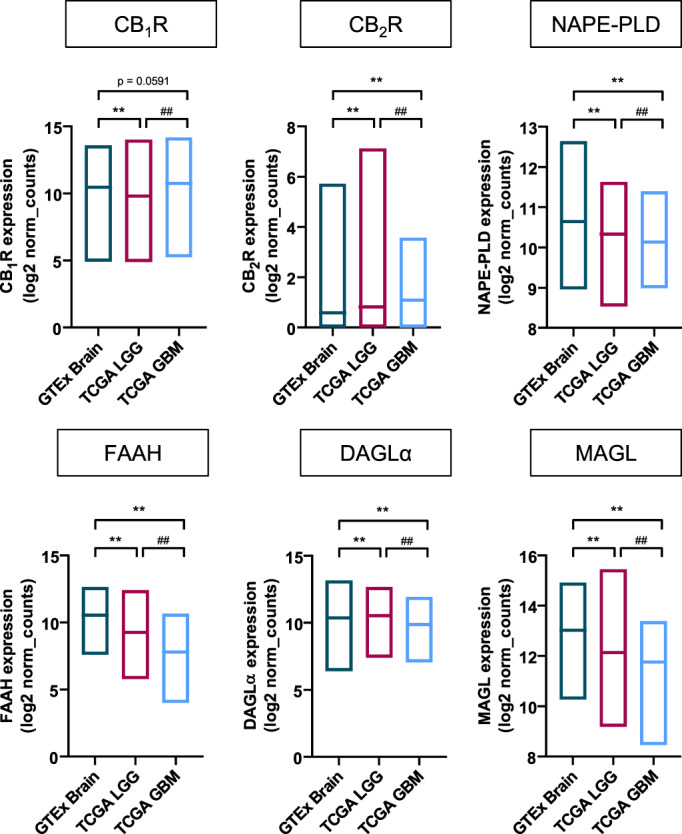
Expression of endocannabinoid system elements in glioma (genomic datasets). mRNA expression (shown as log2 of normalized counts) of different endocannabinoid system elements in specimens from healthy brain tissue (GTEx brain; *n* = 1136), low/medium‐grade gliomas (TCGA LGG; *n* = 523) and glioblastoma (TCGA GBM; *n* = 166). Data were obtained from the cancer genome atlas (TCGA) research network (https://www.cancer.gov/tcga) and the genotype tissue expression (GTEx) portal (https://gtexportal.org/home), and were accessed by using the Xenabrowser tool (http://xena.ucsc.edu). **p < .01 from GTEx brain or ^##^ *p* < .01 from TCGA LGG by one‐way ANOVA with Tukey's post‐hoc test

The recent development of next‐generation sequencing procedures has produced vast amounts of genomic data of samples of basically every disease, including glioblastoma. Public and user‐friendly data‐mining search engines currently empower researchers to easily interrogate freely‐accessible genomic datasets. As a mere proof of concept, here we used the Xena platform (http://xena.ucsc.edu) (Goldman et al., 2020) to compare the mRNA expression of the main components of the ECS between healthy brain, low/medium‐grade gliomas (grades 2–3) (TCGA Research Network, 2015), and glioblastoma (grade 4) (Brennan et al., 2013) by using a combined cohort of The Cancer Genome Atlas (TCGA) (https://www.cancer.gov/tcga) and Genotype Tissue Expression (GTEx) (https://gtexportal.org/home) samples (Figure 1). This approach allowed a very large sample size, which provides sufficient statistical inference power to unveil minor differences between groups. In fact, significant changes between the three groups were detected for every element of the ECS examined, with the sole exception of CB_1_R expression between healthy brain and glioblastoma samples. These differences, both regarding CB_2_R expression and broadly speaking, are similar to the published data discussed above and shown in Table 1. The same trends were observed when comparing with adjacent, non‐tumoral tissue, although in this case the control sample size was too small (*n* = 5) for statistical analyses to be performed with enough confidence. Moreover, according to the Chinese Glioma Genome Atlas (http://www.cgga.org.cn), CB_1_R expression is significantly up‐regulated in IDH‐wild type grade 4 gliomas versus IDH‐mutant grade 4 gliomas (*p* = 2 × 10^−5^), while CB_2_R expression does not change (*p* = .94). We also conducted survival analysis on the 166 samples of the aforementioned TCGA glioblastoma dataset (Brennan et al., 2013), which were stratified for high or low mRNA expression of the different ECS components, as assessed by the Cutoff Finder bioinformatic tool (Budczies et al., 2012). Log‐rank tests on the respective Kaplan–Meier plots only showed a subtle difference (*p* = .0418) in the case of MAGL mRNA, with individuals bearing tumors with low MAGL expression displaying slightly less overall survival than those with high MAGL expression.

## 3 CANNABINOID‐EVOKED GROWTH‐INHIBITING EFFECTS IN GLIOMA CELLS

Since more than 20 years ago (Galve‐Roperh et al., 2000; Sánchez et al., 1998, 2001), a large body of evidence has shown that pharmacological activation of cannabinoid receptors exerts profound effects in glioma cells. Overall, these studies have revealed that natural (THC) as well as synthetic cannabinoid receptor agonists (e.g., the CB_1_R/CB_2_R‐mixed agonists WIN‐55,212‐2 and HU‐210, and the CB_2_R‐selective agonist JWH‐133) evoke growth‐inhibiting effects in glioma cells, both in vitro and upon grafting into host laboratory animals, through the activation of CB_1_R and/or CB_2_R. Nowadays, we know that the mechanism of cannabinoid receptor‐induced anti‐tumoral activity in experimental glioblastoma models is very complex and involves an inhibition not only of cancer cell survival/proliferation, but also of invasiveness, angiogenesis and the stem cell‐like properties of cancer cells, thereby affecting the complex tumor microenvironment (Dumitru et al., 2018; Ellert‐Miklaszewska et al., 2020; Velasco et al., 2012).

**FIGURE 2:**
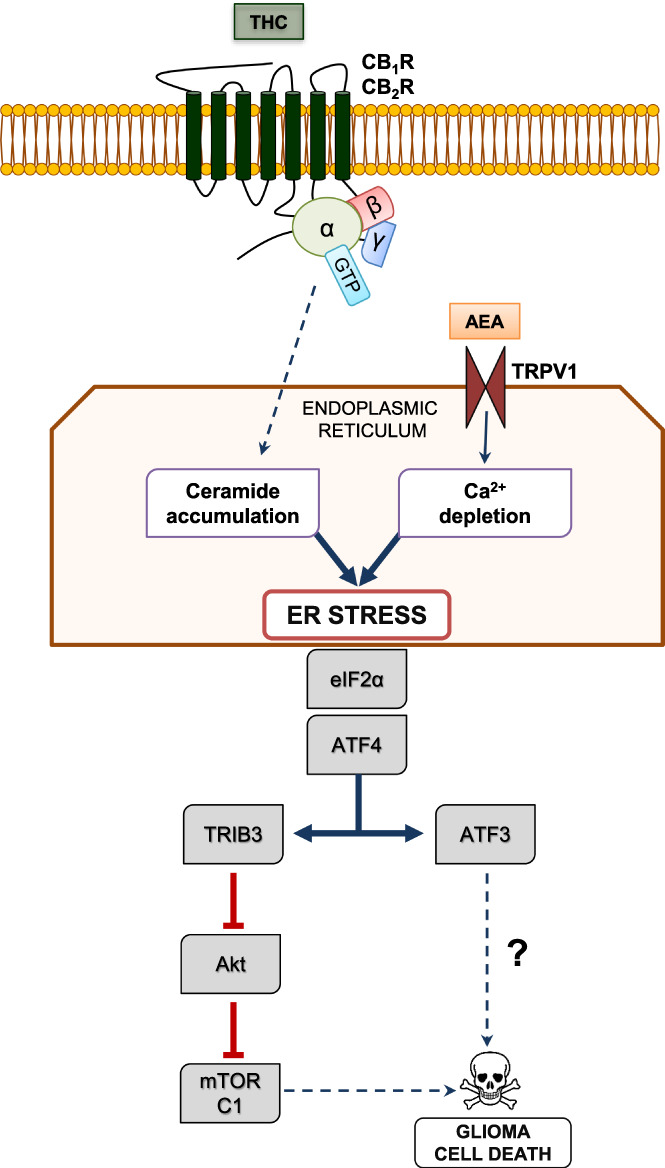
Scheme depicting the mechanism of cannabinoid‐induced apoptosis of glioma cells. Cannabinoids trigger ER stress in glioma cells through at least two mechanisms: (i) THC‐evoked, CB_1_R/CB_2_R‐dependent stimulation of ceramide synthesis de novo in the ER via G protein‐dependent (and perhaps G protein‐independent) actions, and (ii) AEA‐evoked activation of TRPV1 receptors located on the ER, which mediates Ca^2+^ efflux from this organelle to the cytoplasm. Ceramide accumulation and Ca^2+^ depletion in the ER may converge at the phosphorylation (that is, inhibition) of eIF2α and the induction of ATF4, which, in turn, triggers cell death by two pathways: (i) up‐regulation of TRIB3 expression, thereby leading to inhibition of the AKT‐mTORC1 axis and sequential activation of autophagy and apoptosis, and (ii) up‐regulation of ATF3 expression, which induces cell death via a hitherto unknown mechanism. Dashed lines represent multi‐step processes

To date, the best‐established anti‐tumoral effect of THC and other cannabinoid receptor agonists on glioma cells is the induction of apoptosis (Figure 2). Thus, these compounds trigger the apoptotic death of glioma cells by a CB_1_R/CB_2_R‐dependent stimulation of the biosynthesis of the pro‐apoptotic sphingolipid ceramide (Carracedo et al., 2006; Galve‐Roperh et al., 2000). This event occurs in a specific cell organelle, the endoplasmic reticulum (ER), and activates the so‐called ER stress response (Markouli et al., 2020), involving the sequential up‐regulation of the stress‐regulated protein p8, and its downstream targets the transcription factors ATF4 and CHOP (Carracedo et al., 2006). Then, ATF4/CHOP action converges in the expression of TRIB3, a pseudokinase that binds to and inhibits the key pro‐survival protein kinase Akt. Consequently, the Akt substrate mechanistic (formerly “mammalian”) target of rapamycin complex 1 (mTORC1) is inhibited, thereby leading to the stimulation of autophagy (the process of cell “self‐digestion”) and, in turn, of a mitochondrial damage‐mediated pro‐apoptotic response (Cudaback et al., 2010; Salazar et al., 2009). This process of glioma cell death may be accompanied by other CB_1_R/CB_2_R‐evoked cell growth‐inhibiting mechanisms such as the induction of oxidative stress, the blockade of the G1/S cell‐cycle transition, and the regulation of the transcription factor Krox24/Egr1 (Bouaboula et al., 1995; Dumitru et al., 2018; Ellert‐Miklaszewska et al., 2020; Krones‐Herzig et al., 2005; Wang et al., 2021). Additional mechanisms, including the inhibition of angiogenesis (Blázquez et al., 2003) and invasiveness (Blázquez et al., 2008; Ramer & Hinz, 2008), can also contribute to the observed CB_1_R/CB_2_R‐induced impairment of glioma growth in mouse models (see below).

The widely‐reported inhibition of glioma cell proliferation/survival upon cannabinoid receptor stimulation is in striking contrast with the well‐known proliferative/pro‐survival cannabinoid receptor‐dependent activity on neural progenitors, neurons and neuroglial cells. In fact, cannabinoid receptors regulate signal transduction pathways distinctly in tumor and non‐tumor cells. The molecular basis of this “ying‐yang” behavior is incompletely understood yet, but the possibility that it relies on different patterns of CB_1_R/CB_2_R expression and/or pre‐coupling to effectors seems unlikely (discussed in Maccarrone et al., 2014). Hence, cell‐intrinsic downstream molecular features might account for the differences in cannabinoid sensitivity of glioma cells and normal neural cells. For example, tumor and non‐tumor glial cells have a differential capacity to induce de novo ceramide synthesis in the ER upon cannabinoid receptor engagement and, in turn, to trigger an ER stress and pro‐autophagic response, which may determine whether the mitogenic PI3K‐Akt‐mTORC1 pathway becomes inhibited (in glioma cells) or stimulated (in non‐tumor neural cells) (Velasco et al., 2012).

In contrast with this ample information on the pharmacological activation of CB_1_R and CB_2_R, we know little about biological, endocannabinoid‐evoked actions in glioma cells (Ma et al., 2016). A hallmark study (Stock et al., 2012) showed that fatty acyl‐ethanolamides (i.e., AEA and molecular congeners) released from neural progenitor cells of the mouse brain can activate transient receptor potential vanilloid subfamily member‐1 (TRPV1) located on the ER of neighboring glioma cells, thereby inducing Ca^2+^ release into the cytoplasm (Figure 2). This depletes ER Ca^2+^ stores and evokes the phosphorylation/inhibition of eIF2α, and the up‐regulation of transcription factors such as ATF4 and ATF3, leading in turn to glioma cell death, conceivably in convergence with the aforementioned ceramide‐accumulation branch. The molecular targets downstream of ATF3 in the TRPV1‐mediated ER stress response remain however elusive. Of note, systemic administration of arvanil (a synthetic, non‐pungent, blood–brain‐barrier permeable vanilloid) to mice harboring high‐grade gliomas decreased tumor size and extended survival of the animals, thus suggesting a therapeutic potential for TRPV1 agonists (Stock et al., 2012). This process might be retro‐modulated by the reported TRPV1‐evoked attenuation of neural progenitor cell proliferation (Stock et al., 2014). It is also plausible that endocannabinoids encounter additional targets to trigger glioma cell death. For example, 2‐AG inhibits the NF‐κB pro‐inflammatory pathway and cell growth via CB_1_R in human glioma cells (Echigo et al., 2012), and AEA induces apoptosis of human neuroblastoma cells through a CB_1_R‐dependent pathway via MAPKs and BiP/GRP78, an ER stress sensor that up‐regulates p53 and PUMA (Pasquariello et al., 2009).

## 4 EFFECTS OF CANNABINOIDS IN GLIOMA STEM CELLS

Glioblastoma cells exhibit a high degree of cellular and molecular heterogeneity, which renders them reluctant to many therapies, as well as prone to notorious cellular plasticity and tumor recurrency (Wen et al., 2020). The existence of a reduced pool of “glioma stem cells” (GSCs; also known as “glioma‐initiating cells”, GICs) might explain this clinical picture (Bakhshinyan et al., 2021). Although the precise characteristics of the cell of origin of glioblastoma are still under intense debate (Fan et al., 2019), GSCs show remarkable similarities to neural stem cells (NSCs), particularly regarding the expression pattern of stemness genes such as CD133, Sox10, nestin and Musashi (Bakhshinyan et al., 2021). A landmark study has shown that NSCs of the subventricular zone of patients with glioblastoma show driver mutations that match with those found in tumor samples, thus suggesting that aberrant differentiation of NSCs gives rise to glioma (Lee et al., 2018). Of note, a large body of evidence supports a role for the ECS in adult NSC proliferation and differentiation (Galve‐Roperh et al., 2013). Thus, CB_1_R activation induces neuronal differentiation of NSCs (Compagnucci et al., 2013; Jiang et al., 2005), while receptor blockade or genetic ablation reduces it (Hill et al., 2010; Zimmermann et al., 2018). CB_1_R can evoke astroglial differentiation as well (Aguado et al., 2006). CB_2_R also controls proliferation, differentiation and survival of adult NSCs (Downer, 2014; Palazuelos et al., 2006). Likewise, indirect modulation of CB_1_R/CB_2_R by altering the synthetic or degradative enzymes of AEA and 2‐AG leads to a dysregulation of NSCs (Maccarrone et al., 2014; Prenderville et al., 2015). Hence, it is likely that cannabinoid receptors can regulate the function of GSCs. In line with this notion, pharmacological activation of CB_1_R and CB_2_R reduced the potentiality of cultured GSCs, and consequently their tumorigenic potential in vivo, by promoting glial differentiation (Aguado et al., 2007). Moreover, a combination of THC, cannabidiol (CBD) and the alkylating, cytotoxic agent temozolomide inhibited tumor growth in a preclinical model of glioblastoma from GSCs (López‐Valero, Saiz‐Ladera, et al., 2018). More recently, a study has reported that arsenite‐resistance protein 2, a prominent marker of NSCs (Andreu‐Agullo et al., 2012), induces MAGL expression in GSCs, which in turn contributes to enhance self‐renewal and tumorigenicity (Yin et al., 2020). Unfortunately, the authors focused on the production of prostaglandin E2 from arachidonic acid, and the role of 2‐AG was not addressed. As promoting GSC differentiation might be a promising therapy for glioma (Piccirillo et al., 2006; Wang et al., 2017), determining the effects that cannabinoids exert on GSCs in more physiologically‐relevant models constitutes a research niche for the upcoming years (see below).

## 5 EFFECTS OF CANNABINOIDS IN THE GLIOMA MICROENVIRONMENT

Preclinical models of glioblastoma have helped to understand tumor biology and new potential treatment options. These models can be largely grouped in two main categories, namely cell line‐based mouse models and genetically‐engineered mouse models (GEMMs) (Haddad et al., 2021). The classical approach to glioblastoma research involves the grafting of a human glioma cell line (e.g., U87, U251), either subcutaneously or intracranially, in immunodeficient mice. An important advantage of this approach is that it can be adapted to use patient‐derived xenografts (PDXs) aimed to test personalized treatments (Hidalgo et al., 2014). This approach has however several inherent shortcomings such as the use of a homogenous cell population, the occurrence of genetic drift upon cell culture, and the use of mice lacking a functional immune system, which precludes the interaction between immune and cancer cells, a crucial process in neoplastic diseases (Hanahan & Weinberg, 2011). The latter issue may be circumvented by using syngeneic cell lines, that is, cells generated from murine tumors that can therefore by grafted into mice of a similar genetic background (Rall, 1970). Nonetheless, some of these cell lines display a very high mutational burden compared to primary human glioma cells, which might lead to confounding results (Haddad et al., 2021; Hodges et al., 2017). The recent generation of immunodeficient mice with a human‐like immune system might represent an approach to the use of human cell lines instead of syngeneic mouse cell lines (Buqué & Galluzzi, 2018). Unlike cell line‐based mouse models, GEMMs recapitulate the tumor‐generation process, a key step of gliomagenesis. This technology relies on immunocompetent mice, therefore allowing a proper assessment of the tumor microenvironment and enabling crossings between existing cancer‐modeling mouse lines. However, these models, as cell line‐based models, lack significant tumor heterogeneity because tumorigenesis relies on the mutation of only one or a few driver genes.

To date, the anti‐glioma effects of THC and other cannabinoid receptor agonists have been shown upon subcutaneous or intracranial injections of either human or syngeneic glioma cells into immunodeficient mice or immunocompetent rats (Galve‐Roperh et al., 2000; Sánchez et al., 2001; Carracedo et al., 2006; López‐Valero, Torres, et al., 2018;a), thus precluding the study of the precise involvement of cannabinoid receptors in gliomagenesis. Glioblastoma cells can manipulate almost every surrounding cell type to favor tumor development. For example, they are able to boost angiogenesis, recruit astrocytes, evade microglia and macrophages, and even change the neighboring extracellular matrix, to support tumor growth (Broekman et al., 2018). Moreover, a large body of evidence supports a possible role of neuronal activity in the control of glioma progression (Gillespie & Monje, 2018). Unfortunately, the role of cannabinoid receptors residing on cell types within the brain‐tumor microenvironment has not been studied in detail. Nonetheless, just to mention a few possibilities, cannabinoids impair glioma angiogenesis by inhibiting vascular endothelial growth factor production and signaling, as well as by blunting vascular endothelial cell migration and survival (Blázquez et al., 2003, 2004). Likewise, knocking out the FAAH gene causes antiangiogenic effects in vivo (Rieck et al., 2021). Glioblastoma cells can also hijack activated tumor‐associated astrocytes (astrogliosis) to sustain tumor proliferation (O'Brien et al., 2013). As cannabinoids limit astrogliosis in multiple pathological settings (e.g., Aso et al., 2012; Espejo‐Porras et al., 2019; Feliú et al., 2017; Ruiz‐Calvo et al., 2019), it would be plausible that cannabinoid receptor engagement deactivated tumor‐associated astrocytes. In addition, glioma cells express various neurotransmitter receptors and form synapse‐like contacts with neurons, which influences tumor growth (Venkataramani et al., 2019; Venkatesh et al., 2019). In particular, glutamate promotes glioma cell survival, growth and migration through AMPA receptors (Ishiuchi et al., 2002; Takano et al., 2001). As the foremost function of CB_1_R is the inhibitory control of neurotransmission (Piomelli, 2003), anti‐tumoral actions of cannabinoids might conceivably include the blockade of glutamate output by neuron terminals. These and other hypotheses could be tested in the future by using mouse models of loss or gain of function of CB_1_R/CB_2_R and cancer‐driver mutations in selective cell lineages.

## 6 METABOLIC EFFECTS OF CANNABINOIDS IN GLIOMA CELLS

The effects of cannabinoids on cancer cell metabolism have been barely studied to date. High‐grade gliomas show a highly rewired metabolism, similar to many other malignant cancers (Deshmukh et al., 2021). As a general notion, glioma cells display increased glucose and glutamine uptake, enhanced glycolysis, and reduced Krebs cycle (Warburg effect), as well as increased lipogenesis. Although cannabinoid receptors might be conceivably involved in this metabolic reprogramming (see below), the best characterized anti‐tumoral signal evoked by them is Akt inhibition. Akt is a key enzyme in the control of cellular metabolism by growth factors. In fact, PTEN loss, which causes constitutive activation of the PI3K/Akt axis, is a frequent hallmark of glioblastoma. For example, Akt can phosphorylate and stabilize phosphofructokinase P, the main enzyme isoform present in glioma cells, thus favoring glycolysis (Lee et al., 2017). Lipogenesis is also dependent on the epidermal growth factor receptor (EGFR)‐Akt signaling axis, which induces the expression of acetyl‐CoA carboxylase and fatty acid synthase, two committed lipogenic enzymes, as well as net dephosphorylation (and subsequent activation) of acetyl‐CoA carboxylase. To which extent the anti‐tumoral activity of cannabinoids in glioma may include the blockade of glycolysis and lipogenesis is currently unknown. In line with this possibility, attenuation of glycolysis and glutamine uptake has been observed in pancreatic cancer cells upon cannabinoid challenge, thus resulting in cell growth inhibition (Dando et al., 2013), and amphiregulin‐evoked EGFR activation (likely promoting Akt signaling) prevented cannabinoid‐induced apoptosis of glioma cells (Lorente et al., 2009).

Aside from its anti‐tumoral actions, THC has been shown to affect glucose uptake by C6 glioma cells in a biphasic manner, with low doses favoring and high doses inhibiting glucose uptake and lipogenesis (Sánchez et al., 1997). This effect was prevented by the CB_1_R‐selective antagonist rimonabant (SR141716). Unfortunately, a CB_2_R‐selective antagonist was not tested‐though later others showed that CB_2_R stimulation increases glucose uptake in mouse astrocytes and neurons (Köfalvi et al., 2016). How this cannabinoid receptor‐induced glucose uptake and metabolism occurs is not known. Glioma cells express the glucose transporters GLUT1 and GLUT3, whose abundance is up‐regulated by hypoxia‐inducible factors (HIFs) (Kuang et al., 2017; Li et al., 2009). Intriguingly, CB_1_R and CB_2_R knockout mice show diminished GLUT1 expression in the pancreas (Zibolka et al., 2020), and treatment of mouse astrocytes with THC decreases GLUT3 mRNA levels (Jimenez‐Blasco et al., 2020). Thus, control of glucose transporter expression by cannabinoids might be involved in the THC‐modulated glucose uptake by glioma cells. The biphasic profile of THC action on glucose uptake may render a provocative idea. Thus, low (i.e., nanomolar) doses of THC could somewhat reflect a low‐input, “endocannabinoid‐like” activation of cannabinoid receptors, while the cell growth‐inhibiting effects of cannabinoids, that are achieved at higher (i.e., micromolar) doses of THC, would represent a high‐input, “over‐activated” (and rapidly “down‐regulated”?) cannabinoid signaling status. As, for example, (i) low doses of cannabinoid receptor agonists can transactivate EGFR and favor tumor cell growth (Hart et al., 2004) and angiogenesis (Pisanti et al., 2011), (ii) an elevated CB_2_R expression is associated with poor patient prognosis in breast cancer (Pérez‐Gómez et al., 2015), and (iii) specimens of human glioblastoma show augmented levels of 2‐AG and CB_2_R (see above), a pro‐oncogenic role for the ECS seems possible. Accordingly, blocking CB_1_R inhibits the growth of several glioma cell lines (Ciaglia et al., 2015). In (apparent) contrast, anti‐tumoral effects elicited by (high agonist dose‐evoked) engagement of cannabinoid receptors in mouse models of glioma and other cancers have been widely reported, as discussed above (Dumitru et al., 2018; Ellert‐Miklaszewska et al., 2020; Velasco et al., 2012).

Although limited evidence supports to date this “yin‐yang” hypothesis of cannabinoid action on glioma cell growth, effects of cannabinoids in related cell types, such as astrocytes, might provide some ideas. For example, recent evidence shows that mitochondria‐localized CB_1_R (mtCB_1_R) inhibits mitochondrial respiration complex I (Bénard et al., 2012), and astrocytes readily express mtCB_1_R (Gutiérrez‐Rodríguez et al., 2018). Thus, if present in glioma cells, mtCB_1_R might contribute to drive the Warburg effect by inhibiting mitochondrial oxidative metabolism. However, this blockade of mitochondrial complex I in astrocytes also leads to a decrease in ROS production, HIF1 levels, and expression of pro‐tumoral glycolytic and angiogenic genes, thereby hampering lactate production (Jimenez‐Blasco et al., 2020), and so, perhaps, impeding gliomagenesis (Huang et al., 2021). Recently, mtCB_1_R has been shown to enhance Ca^2+^ flux from the ER to mitochondria in astrocytes (Serrat et al., 2021). This mtCB_1_R‐evoked process could operate in concert with AEA‐evoked anti‐glioma action, which relies on Ca^2+^ efflux from the ER upon TRPV1 engagement (Stock et al., 2014). Intriguingly, an intracellularly‐located pool of CB_1_R molecules modulates Ca^2+^ concentration in a neuroblastoma cell line (Brailoiu et al., 2011). Altogether, these various pieces of evidence point to the notion that mtCB_1_R might have a dual role as tumor promoter or tumor suppressor in glioma, likely dependent on subtle, hitherto unknown contextual factors.

## 7 CANNABINOID RECEPTORS AS DRUGGABLE TARGETS FOR GLIOBLASTOMA THERAPY?

The current first‐line strategy for the management of glioblastoma is hardly effective, and relies on the sequential use of surgery, radiotherapy plus concomitant temozolomide, and adjuvant temozolomide ‐usually referred to as “Stupp regime” (Stupp et al., 2005, 2009). Other chemotherapeutic drugs, as well as antibody‐ or gene therapy‐based strategies, have been tested in patients with glioblastoma, but no trial performed to date has been remarkably successful (Wen et al., 2020). It is therefore essential to develop new therapeutic strategies for the management of glioblastoma. The major focus of anticancer therapies has progressively moved from non‐specific chemo‐ and radiotherapies to “personalized”, molecularly‐targeted interventions. In this context, as discussed above, engagement of an unambiguous molecular target (CB_1_R/CB_2_R) by a family of selective compounds (THC and other cannabinoid receptor agonists) efficaciously inhibits the growth of grafted glioblastoma cells in animal (mouse and rat) models through a defined mode of anti‐tumoral action (Luís et al., 2020; Rocha et al., 2014). Preclinical evidence also supports that THC improves the therapeutic efficacy of conventional antineoplastic interventions in glioblastoma [i.e., temozolomide (Torres et al., 2011) and radiotherapy (Scott et al., 2014)]. Moreover, a desirable property of antineoplastic therapies is the preferential targeting of malignant cells. In this regard, THC induces apoptosis of glioblastoma cells with no negative impact on the viability of normal, non‐malignant neural cells (Del Pulgar et al., 2002; Galve‐Roperh et al., 2000; McAllister et al., 2005). Nonetheless, there are important gaps in knowledge that would require future research to optimize cannabinoid receptor‐targeted interventions, for example (i) increasing our understanding of the molecular mechanisms of cannabinoid anti‐tumoral action; (ii) defining the precise biological role of the endocannabinoid system in tumor generation, growth, and progression; (iii) designing the most appropriate cannabinoid‐based combinational therapies in preclinical models of glioblastoma (and other cancers); and (iv) identifying molecular biomarkers of response to cannabinoid anti‐tumoral therapies.

The preliminary clinical testing of cannabinoid anti‐tumoral activity in glioblastoma is currently underway (Abrams et al., 2021; Abrams & Guzmán, 2020). In a pilot Phase 1 study, 9 patients with recurrent glioblastoma underwent intracranial THC administration (Guzmán et al., 2006). Although no statistically‐relevant conclusions could be inferred from such a small cohort, the treatment was safe, and some patients seemed to have responded in terms of reduced tumor growth rate, as evaluated by MRI, and decreased markers of malignancy in tumor specimens. Later, a randomized, double‐blind, placebo‐controlled, Phase 1b study of the oro‐mucosal cannabis extract nabiximols (THC/CBD at 1:1 ratio), added as an adjunct to dose‐intense temozolomide, was conducted in 21 patients with recurrent glioblastoma (Twelves et al., 2021). This study concluded that nabiximols had acceptable safety and tolerability, with no drug–drug interaction identified. In addition, nabiximols seemed to offer some efficacy as an adjunct to chemotherapy as the 1‐year survival rate was 83% (nabiximols group) versus 44% (placebo group) (*p* = .042). The 2‐year survival rate was 50% (nabiximols group) versus 22% (placebo group) (*p* = .134). Concomitantly, a randomized, double‐blind Phase 2 trial of standardized cannabis oils (THC/CBD at 1:1 or 4:1 ratio, *p.o*.) in 88 patients with recurrent or inoperable high‐grade gliomas (Schloss et al., 2021) reported that the 1:1 ratio improved both physical (*p* = .025) and functional (*p* = .014) capacity, as well as sleep (*p* = .009). No serious adverse events occurred. However, no changes in disease progression were found compared to a retrospective‐case group. Finally, the coming years may likely provide valuable data coming from (i) an open‐label Phase 2 trial evaluating the effect of an oral THC/CBD preparation (at 1:1 ratio) concurrently with standard temozolomide‐based chemo‐radiation in 30 patients with newly‐diagnosed glioblastoma (https://clinicaltrials.gov/ct2/show/NCT03529448), and (ii) a randomized, double‐blind, placebo‐controlled, Phase 2 trial evaluating the effect of nabiximols plus temozolomide in 230 patients with recurrent glioblastoma (https://www.thebraintumourcharity.org/media-centre/news/research-news/phase-2-trial-cannabis-based-drug-glioblastomas). Hopefully, these ‐and ideally additional‐controlled, well designed studies will clarify the question of whether cannabinoid‐based therapies could be potentially incorporated into the current pharmacological armamentarium for the management of glioblastoma.

## CONFLICT OF INTEREST

The authors declare no competing financial interests.

## AUTHOR CONTRIBUTIONS

CC‐I and MG wrote the manuscript hand in hand.

## Data Availability

Data sharing is not applicable to this article as no new data were created or analyzed in this study.
